# Skeletal Muscle Insulin Resistance and Absence of Inflammation Characterize Insulin-Resistant Grade I Obese Women

**DOI:** 10.1371/journal.pone.0154119

**Published:** 2016-04-25

**Authors:** Cacylde Amouzou, Cyril Breuker, Odile Fabre, Annick Bourret, Karen Lambert, Olivier Birot, Christine Fédou, Anne-Marie Dupuy, Jean-Paul Cristol, Thibault Sutra, Nicolas Molinari, Laurent Maimoun, Denis Mariano-Goulart, Florence Galtier, Antoine Avignon, Françoise Stanke-Labesque, Jacques Mercier, Ariane Sultan, Catherine Bisbal

**Affiliations:** 1 PhyMedExp, University of Montpellier, INSERM U1046, CNRS UMR 9214, Montpellier, France; 2 Centre Hospitalier Régional Universitaire (CHRU) Montpellier, Montpellier, France; 3 Faculty of Health, York University, York, Ontario, Canada; 4 University of Grenoble Alpes, INSERM U1042, HP2, Grenoble, CHU Albert Michalon, Grenoble, France; INSERM/UMR 1048, FRANCE

## Abstract

**Context:**

Obesity is associated with insulin-resistance (IR), the key feature of type 2 diabetes. Although chronic low-grade inflammation has been identified as a central effector of IR development, it has never been investigated simultaneously at systemic level and locally in skeletal muscle and adipose tissue in obese humans characterized for their insulin sensitivity.

**Objectives:**

We compared metabolic parameters and inflammation at systemic and tissue levels in normal-weight and obese subjects with different insulin sensitivity to better understand the mechanisms involved in IR development.

**Methods:**

30 post-menopausal women were classified as normal-weight insulin-sensitive (controls, CT) and obese (grade I) insulin-sensitive (OIS) or insulin-resistant (OIR) according to their body mass index and homeostasis model assessment of IR index. They underwent a hyperinsulinemic-euglycemic clamp, blood sampling, skeletal muscle and subcutaneous adipose tissue biopsies, an activity questionnaire and a self-administrated dietary recall. We analyzed insulin sensitivity, inflammation and IR-related parameters at the systemic level. In tissues, insulin response was assessed by P-Akt/Akt expression and inflammation by macrophage infiltration as well as cytokines and IκBα expression.

**Results:**

Systemic levels of lipids, adipokines, inflammatory cytokines, and lipopolysaccharides were equivalent between OIS and OIR subjects. In subcutaneous adipose tissue, the number of anti-inflammatory macrophages was higher in OIR than in CT and OIS and was associated with higher IL-6 level. Insulin induced Akt phosphorylation to the same extent in CT, OIS and OIR. In skeletal muscle, we could not detect any inflammation even though IκBα expression was lower in OIR compared to CT. However, while P-Akt/Akt level increased following insulin stimulation in CT and OIS, it remained unchanged in OIR.

**Conclusion:**

Our results show that systemic IR occurs without any change in systemic and tissues inflammation. We identified a muscle defect in insulin response as an early mechanism of IR development in grade I obese post-menopausal women.

## Introduction

Insulin resistance (IR), a metabolic defect associated with obesity, is the key feature of type 2 diabetes (T2D) [1]. Mechanisms leading to IR during obesity are still incompletely understood and are the object of intense research. Several studies performed in humans and rodents reported that a chronic low-grade inflammation at the systemic level as well as within insulin-responding tissues (*e*.*g*. skeletal muscle and white adipose tissue) is the central mechanism leading to IR development in obesity [2–8]. In fact, increased circulating levels of pro-inflammatory mediators such as C-reactive protein (CRP) [9], tumor necrosis factor α (TNFα) [10], monocyte chemoattractant protein-1 (MCP-1) [11] and interleukin-6 (IL-6) [12] have been observed in obesity-associated IR. However, different assumptions coexist in the literature concerning the specific impact of skeletal muscle and/or adipose tissue inflammation and of their infiltration by macrophages in IR pathogenesis in humans.

On the one hand, skeletal muscle is responsible for 85% of glucose uptake and metabolism [13] and, as such, its dysfunction has been considered as a seminal mechanism of IR [14]. Moreover, macrophage accumulation in skeletal muscle has been associated with IR both in mice and humans [4, 15]. On the other hand, white adipose tissue is an endocrine organ that plays an important role in the regulation of whole-body metabolism [5, 16]. Its remodeling and dysfunction induced by obesity have been described as essential contributors to insulin sensitivity impairment [17]. In subcutaneous adipose tissue (SAT), non-esterified fatty acids (NEFA) and lipopolysaccharides (LPS) from the gut microbiota [18, 19] might activate toll-like receptors (TLR) such as TLR4. TLR4 activation induces nuclear factor of kappa light polypeptide gene enhancer in B-cells (NFκB) activation and the secretion of inflammatory cytokines by adipocytes. This leads to a switch in infiltrated macrophages from the alternative M2 anti-inflammatory into the classical M1 pro-inflammatory M1 phenotype and the inhibition of insulin signaling [2]. Indeed, obesity and systemic IR have been associated with an increased proportion of M1 macrophages in SAT [20], which promotes fibrosis and changes in synthesis and release of adipokines involved in insulin signaling regulation [21, 22]. All these studies have contributed to establish that a chronic low-inflammatory state combined with SAT dysfunction could play a critical role in IR development [23–26].

Despite these different observations, inflammation and IR have never been investigated simultaneously at both the systemic level and locally in skeletal muscle and SAT of obese humans. Given that systemic insulin sensitivity is preserved in some obese individuals [27], we compared obese insulin-resistant (OIR), obese insulin-sensitive (OIS), and normal-weight insulin-sensitive subjects (controls, CT). We analyzed key factors previously described as essential mediators of IR development focusing especially on inflammation, both at the systemic level and locally in skeletal muscle and SAT. We also characterized these subjects for various metabolic parameters and lifestyle factors such as their level of physical activity and eating behavior. Our results provide evidence that OIR skeletal muscle becomes insulin-resistant when SAT is still insulin-sensitive and before any obvious sign of inflammation at the systemic or tissue level.

## Materials and Methods

### Study population

We recruited 10 normal-weight (18.5 kg/m^2^ <body mass index (BMI) <25.0 kg/m^2^) and 20 obese (grade I obesity, BMI 30.0–34.9 kg/m^2^) post-menopausal women, aged between 50 and 64 years. They had had a stable weight for at least six months and had no personal or familial history of diabetes, no inflammatory pathology (high-sensitivity C-reactive protein (hs-CRP)<7 mg/mL), no smoking habits and no treatment that could interfere with insulin sensitivity. Their physical activity level was estimated using Voorrips questionnaire [28]. Dietary intakes were assessed by self-administered questionnaire in which the subjects indicated the amount of each beverage and food they had drunk and eaten and the cooking method [29]. These data were reported during three consecutive days, including one Saturday or Sunday to take into account potential different food habits during the weekend. Questionnaires were then analyzed using GENI software (Micro6, Villiers-lès-Nancy, France). Results are expressed as calorie intake (Kcal) and quantity of proteins, fat and carbohydrates in grams per day. Blood pressure was measured after a 15-min rest period using an automated device.

Insulin sensitivity was first assessed by the homeostasis model assessment of IR (HOMA_IR_) index in order to classify the subjects in CT, OIS (HOMA_IR_<2.7) and OIR (HOMA_IR_>2.7) groups [30].

All clinical investigations have been conducted according to the principles expressed in the Declaration of Helsinki. The study protocol was approved by the local Ethic Committee of CHRU Montpellier (Comité de Protection des Personnes Sud-Méditerranée IV) (number 2011.01.04) and informed written consent was obtained from all the participants. The trial was registered on *ClinicalTrials*.*gov* (NCT01561664).

### Body composition

Body composition was assessed by dual-energy X-ray absorptiometry. Visceral adiposity index (VAI) and body adiposity index (BAI) were calculated by the following mathematical formulas, as previously described [31, 32]: VAI = (waist circumference/36.58+(1.89xBMI))x(TG/0.81)x(1.52/HDL) and BAI = [waist circumference/(height)^1.5^]-18).

### Hyperinsulinemic-euglycemic clamp

All subjects underwent a hyperinsulinemic-euglycemic clamp [33] to determine their glucose infusion rate (GIR). First, a bolus insulin dose (6 mIU/kg/min) was administrated for an initial 1 min; thereafter the subjects received a continuous 1 mIU/kg/min insulin infusion for 120 min, as previously described [29]. The GIR index was calculated during the final 30 min of the clamp and expressed as mg/kg lean mass/min [34].

### Biological analyses

Plasma insulin concentration was determined by radioimmunoassay (BI-INS-IRMA kit, Cisbio Bioassays, Codolet, France), plasma glucose concentration using the glucose oxidase method (AU2700 Olympus, Beckman Coulter, Villepinte, France) and NEFA using an enzymatic colorimetric method assay (Wako, Neuss, Germany). Hepatic enzymes, *i*.*e*. aspartate aminotransferase (AST), alanine aminotransferase (ALT) and gamma-glutamyl transpeptidase (GGT), as well as total cholesterol, HDL-cholesterol (HDLc) and triglyceride levels were determined using spectrophotometric methods (AU2700 Olympus, Beckman Coulter). LDL-cholesterol (LDLc) was then calculated using the Friedewald formula. Glycated hemoglobin HbA1c was analyzed by routine high-performance liquid chromatography (HPLC)-based ion-exchange procedure (HA-8140; Menarini, Rungis Cedex, France). hs-CRP was determined by immunoturbidimetry [35], plasma concentrations of cytokines using Biochip Array Technology (Evidence Investigator analyser, Cytokine array I and High Sensitivity, Randox Laboratories, Antrim, UK), and leptin, adiponectin, and resistin concentrations using enzyme-linked immunosorbent assay (ELISA, R&D Systems, Inc., Minneapolis, USA). Plasma concentration of LPS was evaluated by ELISA-based Endotoxin Detection Assay (Hyglos GmbH, Bernried am Starnberger See, Germany). LPS-binding protein (LBP), soluble cluster of differentiation 14 (sCD14) and fetuin-A were determined using ELISA (Hycult biotech, Uden, The Netherlands for LBP, and BioVendor, Brno, Czech Republic for sCD14 and fetuin-A). All these parameters were measured in fasting blood samples.

### Tissue biopsies

Skeletal muscle and SAT biopsies were respectively obtained from the left *vastus lateralis* [36] and periombilical subcutaneous fat area [37] at rest and in fasting state, after local anesthesia with xylocaine 1%.

### Immunohistochemical analysis of muscle and subcutaneous adipose tissue biopsies

Skeletal muscle samples were frozen in cooled isopentan and 10-μm cryosections were performed. SAT samples were formalin-fixed, paraffin-embedded and 4-μm sections were performed [38]. Tissue sections were fixed in 4% formaldehyde and double-stained with anti-CD68 (marker of total macrophage fraction) (Dako, les Ulis, France) and anti-CD86 (marker of M1 pro-inflammatory macrophages) or anti-CD68 and anti-CD206 (marker of M2 anti-inflammatory macrophages) (Santa Cruz Biotechnology, Heidelberg, Germany) primary antibodies, which were used at a 1:100 dilution in PBS/10% fetal bovine serum. Secondary antibodies were anti-rabbit Alexa fluor 488 and anti-mouse Alexa fluor 594 (ThermoFisher Scientific, Sankt Leon-Rot, Germany), and were used at a 1:1000 dilution in PBS/10% fetal bovine serum. Nuclei were stained by 4',6-diamidino-2-phenylindole (DAPI) (Sigma-Aldrich, Lyon, France), used at a 1:2000 dilution in Mowiol mounting medium (2.4 g Mowiol 4–88 from Sigma-Aldrich, 6 g glycerol, 6 mL distilled water, 12 mL 0.2 M Tris-Cl buffer· pH 8.5). Adipocyte size was estimated using ImageJ software (National Institutes of Health, USA, available online at http://rsbweb.nih.gov/ij/index.html). Muscle and SAT sections were observed with a Zeiss AxioImager M1 microscope (Zeiss, Le Pecq, France) and TIFF images were captured with an Axiocam MRm Zeiss using Axiovision 4.7 (05–2008) software.

### Analysis of muscle and adipose tissue proteins

Insulin response was assessed in adipose tissue and skeletal muscle by measuring P-Akt/Akt protein *ratio* in biopsies incubated or not with insulin, as previously described [29, 39–41]. Two 50-mg pieces of fresh skeletal muscle or two 100-mg pieces of fresh abdominal SAT were washed in phosphate buffer saline (PBS). Then, one entire explant was incubated at 30°C (skeletal muscle) or 37°C (SAT) for 15 min with PBS only or supplemented with 1 μM human insulin (Umuline RAPIDE; Lilly Neuilly-sur-Seine, France) [29]. The explants were then disrupted and homogenized in a hypotonic lysis buffer [42] with protease and phosphatase inhibitors (Sigma-Aldrich). Protein concentration was measured in the extracts using the Pierce BCA Protein Assay Kit (ThermoFisher Scientific). Protein expression was assessed on 40-μg (skeletal muscle) or 20-μg (SAT) protein extracts by western blot, using antibodies diluted in Odyssey Blocking Buffer (LI-COR Biosciences, Bad Homburg, Germany). Insulin response was determined using anti-Phospho-Akt (P-Akt) (Ser473) and anti-Akt antibodies at a 1:1000 dilution (both from Cell Signaling Technology Inc., Danvers, USA). Similarly, IκBα protein expression was analyzed in skeletal muscle and SAT using anti-IκBα primary antibody at a 1:500 dilution (Cell Signaling Technology Inc.). All membranes were also incubated with α-β-tubulin antibody at a 1:10000 dilution (Cell Signaling Technology Inc.). Following primary antibody incubation, nitrocellulose membranes were incubated with species-directed secondary antibodies conjugated to IRDye800, at a 1:30000 dilution (Rockland Immunochemicals, tebu-bio, Le Perray-en-Yvelines, France). Specific bands were visualized with a LI-COR Odyssey CLx Imaging System (LI-COR Biosciences). Protein expression levels were quantified using ImageJ software. Finally, levels of protein expression in each sample were corrected using the quantification of the corresponding loading control (Akt for P-Akt or α/β-tubulin for IκBα) analyzed on the same membrane.

### Total RNA purification, cDNA synthesis and quantitative real-time polymerase chain reaction (qPCR)

Total RNA was isolated from skeletal muscle (50 mg) and SAT (100 mg) samples using RNeasy Fibrous Tissue and RNeasy Lipid Tissue Mini Kits (Qiagen Sciences, Courtabœuf, France), respectively. cDNA was generated from 1 μg RNA by reverse transcription using the Verso cDNA kit (ThermoFisher Scientific) in incubation volume of 20 μl. cDNA amplification was then performed by real-time qPCR using the LightCycler^®^ 480 SYBR Green I Master kit (Roche Diagnostics Limited, Rotkreuz, Switzerland) and specific primers for sequences of interest (*i*.*e*. **Col5A: forward** TTGTGGCTCTCTTGTGGTG, **reverse** CAGTGGAAAGGAAAGTGACG; **Col6A: forward** AAATGTGCTCTTGCTGTGAA, **reverse** GCATCTGGCTGTGGCTGTA; **TNFα: forward** TCTTCTCCTTCCTGATCGTG, **reverse** TTGAGGGTTTGCTACAACAT; **IL-1β: forward** CTCCTTTCAGGGCCAATC, **reverse** GGAAGCGGTTGCTCATC; **IL-6: forward** CTTCAGAACGAATTGACAAA, **reverse** CAGGCAAGTCTCCTCATT; **MCP-1: forward** CTCAGCCAGATGCAATCAATG, **reverse** GAGTTTGGGTTTGCTTGTCC; **eEF1α: forward** CATGTGTGTTGAGAGCTTC, **reverse** GAAAACCAAAGTGGTCCAC) (Eurofins Scientific, Ebersberg, Germany). qPCR mix was composed as followed: 2 μL of 1:10-diluted cDNA (10 ng), 0.5 μL of each primer (0.5 μM final concentration), 5 μL 2X concentrated LightCycler^®^ 480 SYBR Green I Master and 2 μL PCR Grade water for a final volume of 10 μL. Relative mRNA expression was quantified according to the comparative cycle threshold method [43], using Ct values in the formula 2^[Ct target gene—Ct reference gene]^ (2^ΔCt^) with eEF1α (eukaryotic translation elongation factor 1 alpha) as stable reference gene [44].

### Statistical analysis

The non-normal distribution of our data was assessed with the Shapiro-Wilk test. Mann-Whitney tests were performed for group comparison according to the non-normal distribution of our data. Wilcoxon test was used for paired sample comparison (basal and insulin-stimulated P-Akt/Akt levels). We considered that significance was reached when P≤0.05. Correlations were determined by Spearman analysis. Statistical tests were performed using R software (version 3.1.0, 2014-04-10, The R Foundation for Statistical Computing, Vienna, Austria).

## Results

### Clinical and biological characteristics of the subjects

All subjects were of equivalent age (Table 1). Obese subjects, regardless of whether they were insulin-sensitive or insulin-resistant, had moderate grade I obesity and had equivalent BMI, total fat mass, body adiposity index, visceral adiposity index. Waist circumference was also alike and positively correlated to HOMA_IR_ (R = 0.741; P<0.0001). Subjects were classified as normal-weight insulin-sensitive (controls, CT), obese insulin-sensitive (OIS) or obese insulin-resistant (OIR), depending on their BMI and HOMA_IR_ index [30]. The greater HOMA_IR_ in OIR subjects was due to greater fasting insulinemia, while fasting glycaemia was in the normal range. GIR measurements during the hyperinsulinemic-euglycemic clamp confirmed lower peripheral insulin sensitivity in OIR subjects compared to OIS and CT at the systemic level (Table 1). Moreover, we observed a highly significant correlation between HOMA_IR_ and GIR indexes (R = -0.577, P = 0.001).

**Table 1:** Clinical and biological characteristics of the subjects.

	CT (n = 10)	OIS (n = 11)	OIR (n = 9)	P-values
**Age (years)**	55.6 ± 3.3	58.0 ± 4.4	55.5 ± 3.8	NS
**Voorrips score**	6.4 ± 2.4	3.0 ± 1.9	3.2 ± 2.3	P_OIS *vs* CT_ = 0.007 P_OIR *vs* CT_ = 0.01
**BMI (kg/m**^**2**^**)**	22.3 ± 1.9	32.0 ± 1.5	33.3 ± 1.8	P_OIS *vs* CT_<0.001 P_OIR *vs* CT_<0.001
**Waist circumference (cm)**	75.6 ± 5.7	96.9 ± 11.0	105.3 ± 8.5	P_OIS *vs* CT_<0.001 P_OIR *vs* CT_<0.001
**Total fat mass (kg)**	20.4 ± 3.9	36.3 ± 4.3	36.3 ± 5.0	P_OIS *vs* CT_<0.001 P_OIR *vs* CT_<0.001
**Body adiposity index**	28.2 ± 2.2	36.9 ± 2.9	37.5 ± 3.6	P_OIS *vs* CT_<0.001 P_OIR *vs* CT_<0.001
**Visceral adiposity index**	0.86 ± 0.3	1.63 ± 1.0	1.95 ± 1.0	P_OIS *vs* CT_ = 0.01 P_OIR *vs* CT_ = 0.003
**Systolic blood pressure (mmHg)**	116.7 ± 14.7	130.6 ± 19.3	132.4 ± 13.0	P_OIR *vs* CT_ = 0.02
**Diastolic blood pressure (mmHg)**	66.5 ± 11.6	75.5 ± 12.6	83.3 ± 13.6	P_OIR *vs* CT_ = 0.02 P_OIR *vs* OIS_ = 0.05
**Fasting glycaemia (mmol/L)**	5.0 ± 0.6	4.9 ± 0.3	5.3 ± 0.6	NS
**Fasting insulinemia (μIU/mL)**	4.9 ± 1.7	8.2 ± 2.8	17.4 ± 3.9	P_OIS *vs* CT_ = 0.006 P_OIR *vs* CT_<0.0001 P_OIR *vs* OIS_<0.0001
**HOMA**_**IR**_	1.1 ± 0.3	1.7 ± 0.6	4.0 ± 0.8	P_OIS *vs* CT_ = 0.006 P_OIR *vs* CT_<0.001 P_OIR *vs* OIS_<0.001
**GIR (mg/kg lean mass/min)**	6.8 ± 2.6	7.0 ± 2.4	4.6 ± 0.6	P_OIR *vs* CT_ = 0.04 P_OIR *vs* OIS_ = 0.01
**HbA1c (mmol/mol)**	39.4 ± 3.1	37.0 ± 4.1	39.8 ± 3.5	NS
**Total cholesterol (mmol/L)**	5.5 ± 0.8	5.9 ± 0.8	5.9 ± 0.7	NS
**HDL-cholesterol (mmol/L)**	2.0 ± 0.5	1.5 ± 0.4	1.6 ± 0.2	P_OIS *vs* CT_ = 0.01
**LDL-cholesterol (mmol/L)**	3.1 ± 0.7	3.9 ± 0.7	3.7 ± 0.6	P_OIS *vs* CT_ = 0.05
**Triglycerides (mmol/L)**	0.8 ± 0.2	1.2 ± 0.4	1.5 ± 0.8	P_OIS *vs* CT_ = 0.03 P_OIR *vs* CT_ = 0.01
**AST (IU/L)**	21.3 ± 6.2	17.6 ± 3.5	21.0 ± 3.5	P_OIS *vs* CT_ = 0.04 P_OIR *vs* OIS_ = 0.03
**ALT (IU/L)**	19.2 ± 7.3	17.4 ± 7.1	26.3 ± 7.8	P_OIR *vs* CT_ = 0.02 P_OIR *vs* OIS_ = 0.009
**GGT (IU/L)**	24.3 ± 15.8	25.5 ± 13.3	23.0 ± 9.8	NS

P-values were obtained using Mann-Whitney tests. Only P-values indicating a statistically significant difference (P≤0.05) between two groups were reported in the table. NS (non-significant) indicates no statistically significant difference between the groups. For all characteristics, data are indicated as mean ± SD.

**Fig 1:**
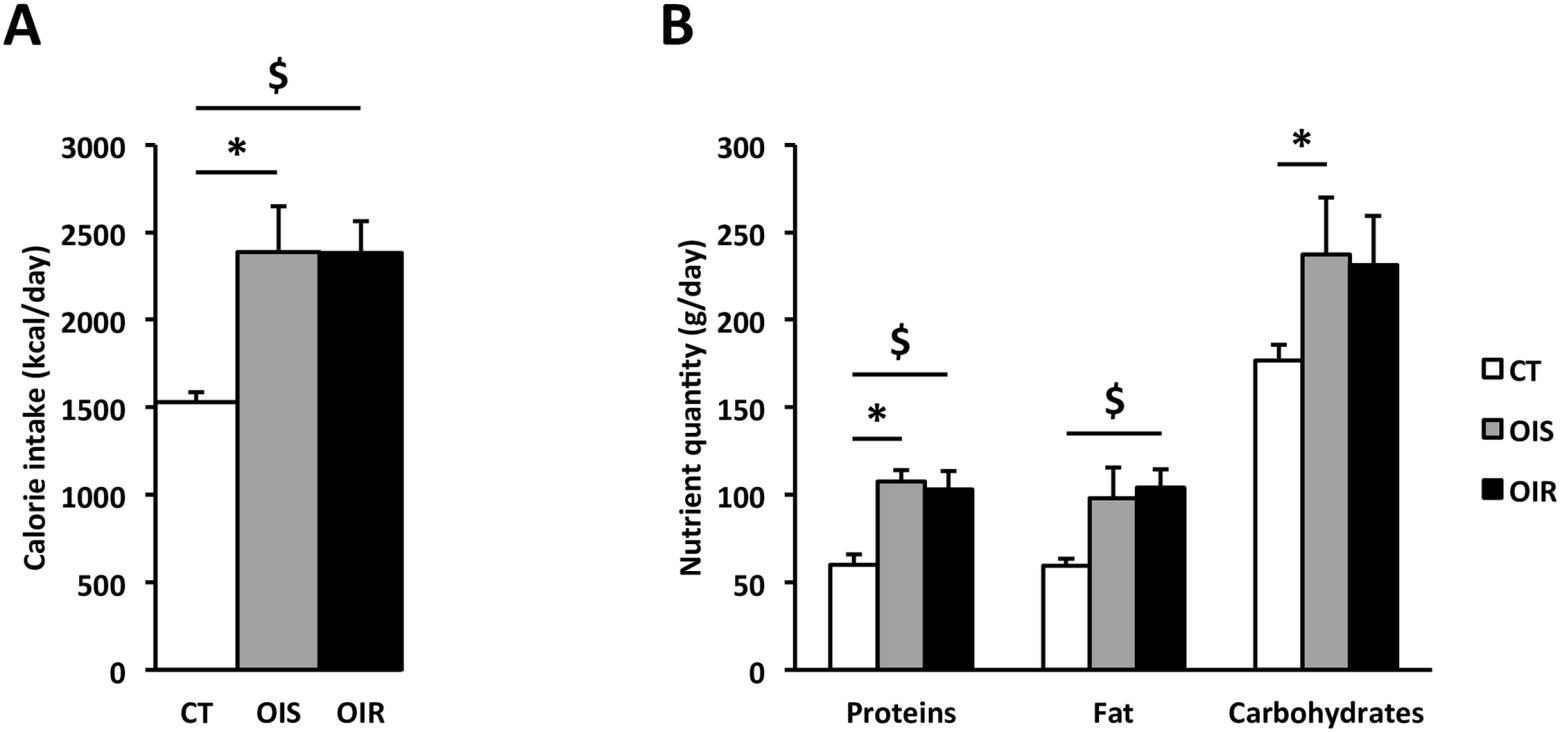
Dietary intake. Calorie intake (**Panel A**) and diet composition (**Panel B**) were determined by self-administered questionnaire and analysis using GENI software. Data are expressed as mean ± SEM. Calorie, * P_OIS *vs*. CT_ = 0.0007; $ P_OIR *vs*. CT_ = 0.0003. Proteins, * P_OIS *vs*. CT_ = 0.0007; $ P_OIR *vs*. CT_ = 0.009. Fats, $ P_OIR *vs*. CT_ = 0.0003. Carbohydrates, * P_OIS *vs*. CT_ = 0.04.

Regarding lifestyle-related parameters, OIS and OIR subjects had close physical activity levels that were lower compared to CT subjects (Voorrips score, Table 1). Their calorie, protein, fat, and carbohydrate intakes were also greater compared to intakes of CT subjects (Fig 1A and 1B). Regarding body composition, measurement of the waist circumference and of the visceral adiposity index indicated that OIS and OIR groups presented the same level of central/visceral adiposity (Table 1).

HbA1c, total cholesterol, and GGT levels were similar in the three groups. Moreover, systolic blood pressure, HDLc, LDLc and triglyceride levels were not different between OIS and OIR confirming similarities in their phenotype (Table 1). Diastolic blood pressure and ALT plasmatic level were slightly higher in OIR group compared to OIS and CT. Regarding adipokine plasmatic concentrations, we observed greater leptin and lower adiponectin levels as well as a tendency for lower ghrelin levels (P_OIR *vs*. CT_ = 0.09) in obese subjects compared to CT, without any difference between OIS and OIR subjects (Table 2).

**Table 2:** Levels of adipokines and markers of systemic inflammation.

	CT (n = 10)	OIS (n = 11)	OIR (n = 9)	P-values
**Leptin (ng/mL)**	14.7 ± 10.6	44.6 ± 13.7	44.7 ± 14.3	P_OIS *vs* CT_<0.001 P_OIR *vs* CT_ = 0.001
**Adiponectin (μg/mL)**	17.4 ± 8.7	12.1 ± 5.2	9.3 ± 3.3	P_OIR *vs* CT_ = 0.03
**Resistin (ng/mL)**	5.0 ± 1.6	5.4 ± 2.0	5.7 ± 2.9	NS
**Ghrelin (pg/mL)**	43.5 ± 17.0	35.4 ± 17.0	29.9 ± 17.4	NS
**hs-CRP (mg/L)**	0.9 ± 0.9	2.1 ± 1.1	2.5 ± 2.4	P_OIS *vs* CT_ = 0.008 P_OIR *vs* CT_ = 0.05
**TNFα (pg/mL)**	1.8 ± 0.9	1.6 ± 0.4	1.8 ± 0.4	NS
**MCP-1 (pg/mL)**	244.4 ± 58.0	227.5 ± 61.7	309.9 ± 81.0	NS
**IFNγ (pg/mL)**	1.05 ± 0.75	1.15 ± 0.77	1.49 ± 0.79	NS
**IL-1α (pg/mL)**	0.32 ± 0.13	0.27 ± 0.14	0.63 ± 0.95	NS
**IL-1β (pg/mL)**	0.80 ± 0.46	0.76 ± 0.41	0.82 ± 0.36	NS
**IL-2 (pg/mL)**	3.05 ± 1.11	3.58 ± 0.89	2.89 ± 1.19	NS
**IL-4 (pg/mL)**	1.83 ± 0.46	2.20 ± 0.67	1.95 ± 0.54	NS
**IL-6 (pg/mL)**	1.25 ± 1.53	1.33 ± 0.84	1.57 ± 1.14	NS
**IL-8 (pg/mL)**	10.22 ± 6.64	12.62 ± 9.44	10.64 ± 8.27	NS
**IL-10 (pg/mL)**	1.13 ± 0.29	0.91 ± 0.32	0.97 ± 0.36	P_OIS *vs* CT_ = 0.04
**LPS (EU/mL)**	0.49 ± 0.17	0.54 ± 0.21	0.51 ± 0.31	NS
**NEFA (mmol/L)**	0.76 ± 0.44	0.64 ± 0.14	0.73 ± 0.38	NS
**sCD14 (μg/mL)**	10.88 ± 2.10	10.69 ± 1.81	10.07 ± 1.68	NS
**LBP (μg/mL)**	11.13 ± 4.19	12.58 ± 4.10	12.97 ± 3.64	NS
**Fetuin-A (μg/mL)**	336.1 ± 74.8	333.8 ± 67.4	391.1 ± 68.9	P_OIR *vs* OIS_ = 0.01

P-values were obtained using Mann-Whitney tests. Only P-values indicating a statistically significant difference (P≤0.05) between two groups were reported in the table. NS (“no significant”) indicates no statistically significant difference between the groups. For all characteristics, data are indicated as mean ± SD.

### Systemic inflammatory markers

Systemic concentrations of well-known markers of inflammatory response are presented in Table 2. hs-CRP levels were greater in obese subjects compared to CT, but were equivalent between OIR and OIS. In line with this observation, hs-CRP levels were positively correlated with BMI (R = 0.576, P<0.0001) and waist circumference (R = 0.502, P = 0.005). We did not observe any difference in systemic pro-inflammatory cytokine levels or in systemic anti-inflammatory IL-4 level between CT, OIS and OIR groups. The levels of the anti-inflammatory IL-10 was slightly lower in OIS subjects compared to CT but was not different between OIS and OIR subjects (Table 2).

We further measured systemic levels of inducers of inflammation: NEFA and LPS. Plasmatic levels of NEFA and LPS were equivalent in the three groups of subjects (Table 2). Therefore, we measured systemic levels of LPS binding protein (LBP) and soluble cluster of differentiation 14 (sCD14), known to enable interaction between LPS and the signaling receptor complex lymphocyte antigen 96 (MD-2)/TLR4 [45] (Table 2). We also measured fetuin-A, recently identified as an endogenous ligand of TLR4 and essential in NEFA-induced IR [46] (Table 2). Only fetuin-A concentration was slightly but significantly greater in OIR group compared to OIS (Table 2).

### Absence of inflammation and maintained insulin sensitivity in SAT from OIR subjects

**Fig 2:**
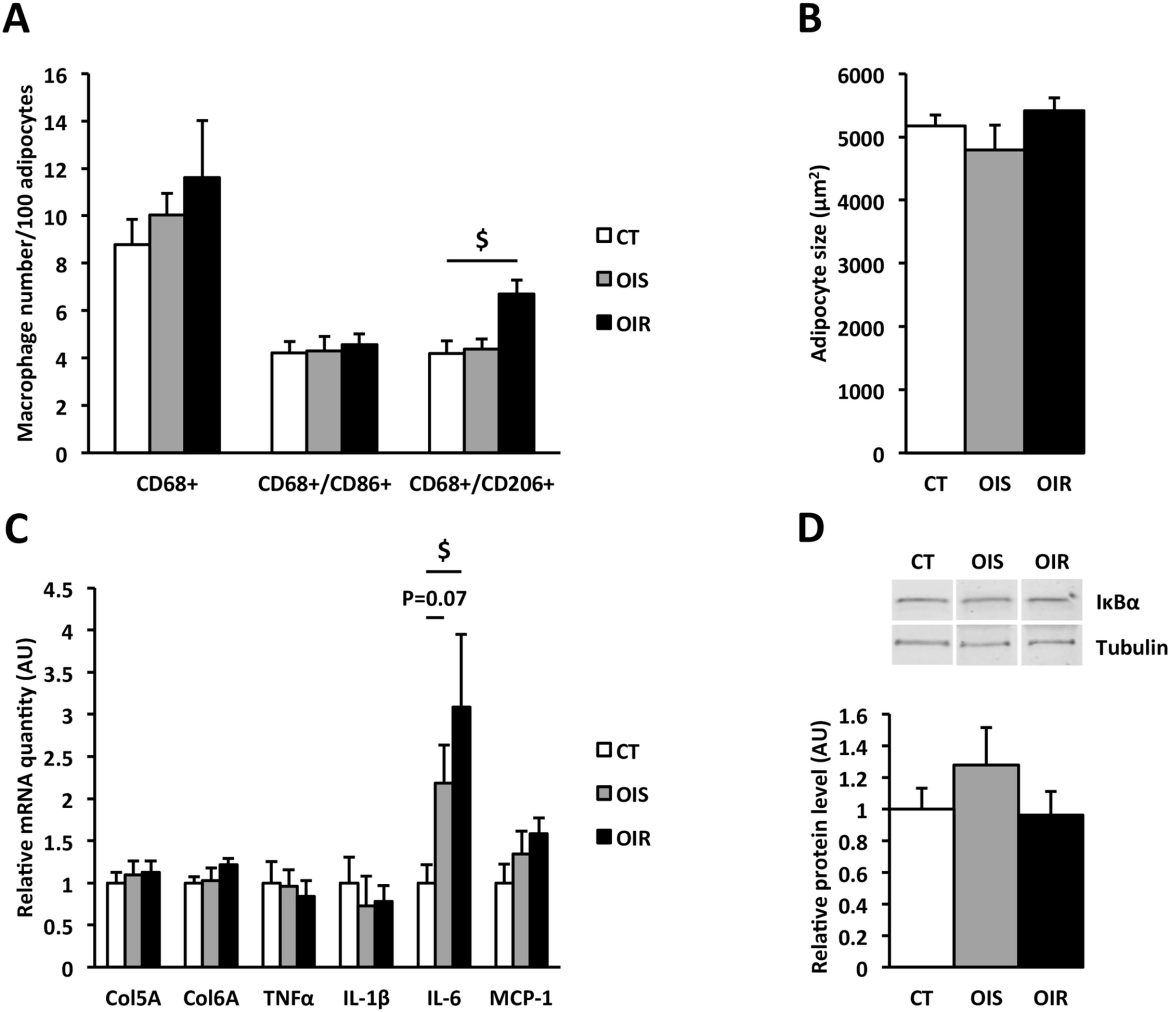
Inflammation and fibrosis in SAT. **Panel A:** Total macrophages (CD68^+^), pro-inflammatory (CD68^+^/CD86^+^), and anti-inflammatory (CD68^+^/CD206^+^) macrophages were counted after staining in SAT. For CD68, and CD86 or CD206 markers, four and two non-consecutive entire sections per subject were analyzed for 7 CT, 5 OIS and 6 OIR subjects. Data are expressed as mean ± SEM. $: P_OIR *vs*. CT_ = 0.008. **Panel B:** Adipocyte average size (μm^2^) in SAT; 10–15 sections per subject were respectively analyzed for 4 subjects per group. Data are expressed as mean ± SEM. **Panel C:** Relative quantification of Col5A, Col6A, TNFα, IL-1β, IL-6 and MCP-1 mRNA expression in SAT (n_CT_ = 10, n_OIS_ = 11, n_OIR_ = 9). Data are expressed as mean ± SEM and relatively to CT values, which were set at 1.0. $ P_OIR *vs*. CT_ = 0.004. **Panel D:** Western blot analysis of IκBα expression in SAT (n_CT_ = 9, n_OIS_ = 8, n_OIR_ = 8). The graph represents IκBα protein quantification after correction by α/β tubulin protein levels, used as an indicator of protein loading. Data are expressed as mean ± SEM and relatively to CT values, which were set at 1.0.

**Fig 3:**
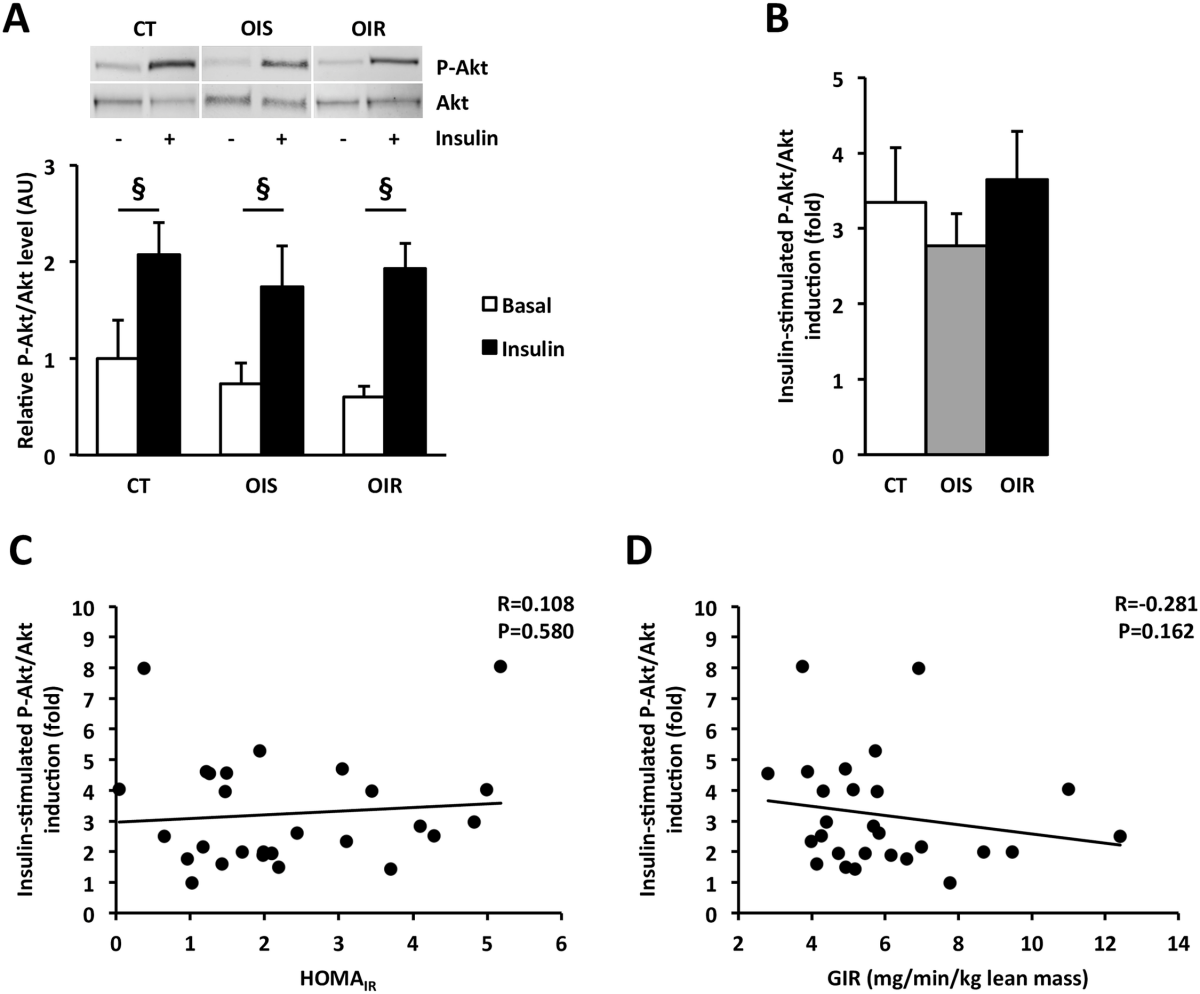
Insulin response in SAT. **Panel A:** Western blot analysis of P-Akt/Akt *ratio* in SAT with and without incubation with insulin (n_CT_ = 9, n_OIS_ = 10, n_OIR_ = 9). The graph represents P-Akt protein quantification after correction by total Akt protein levels, used as an indicator of protein loading. Data are expressed as mean ± SEM and relatively to CT values, which were set at 1.0. § P_CT±insulin_ = 0.008; P_OIS±insulin_ = 0.002; P_OIR±insulin_ = 0.004. **Panel B:** Fold induction of P-Akt/Akt level in SAT after insulin stimulation. Data are expressed as mean ± SEM. **Panels C and D:** Correlations (Spearman analysis) between insulin-stimulated P-Akt/Akt fold induction in SAT and HOMA_IR_ (C) or GIR (D).

SAT dysfunction and inflammation, characterized by adipocyte hypertrophy, fibrosis, progressive tissue infiltration by immune cells, and cytokine secretion, have been described as early events leading to SAT and systemic IR [2, 10, 11]. We first investigated whether inflammation developed locally within SAT. Staining and counting of the different types of macrophages indicated no difference in total macrophage (CD68^+^) number and in the pro-inflammatory macrophage (CD68^+^/CD86^+^) number between CT, OIS and OIR SAT (Fig 2A and S1 Fig). We failed to detect pro-inflammatory macrophages with the particular phenotype CD206^+^/CD11c^+^, previously described as associated with IR in human obesity [47] (data not shown). Anti-inflammatory macrophage (CD68^+^/CD206^+^) number was greater in OIR SAT (P_CT *vs*. OIR_ = 0.008) when compared to CT (Fig 2A). Some studies reported that insulin sensitivity is inversely correlated with average subcutaneous abdominal adipocyte size [48]. However, we did not observe any difference in average adipocyte size between the three groups (Fig 2B). In the same way, SAT fibrosis with collagen accumulation has been associated with SAT IR [49, 50]. We thus examined the expression of two components of the extracellular matrix, *i*.*e*. type V and type VI collagens (Col5A and Col6A). Again, we could not detect any modification in Col5A and Col6A mRNA expression between the three groups (Fig 2C). We also measured mRNA expression of several pro-inflammatory (TNFα, IL-1β, IL-6, MCP-1) cytokines in SAT. TNFα, IL-1β and MCP-1 mRNA expression was equivalent in the three groups whereas IL-6 mRNA expression was greater in OIR SAT compared to CT (P_OIR *vs*. CT_ = 0.004) (Fig 2C) with also a tendency to be higher in OIS SAT (P_OIS *vs*. CT_ = 0.07). Interestingly, IL-6 mRNA levels were positively correlated with anti-inflammatory macrophage (CD68^+^/CD206^+^) number (R = 0.564, P = 0.015). As an indicator of SAT inflammation, we also investigated the activation of TLR4 pathway by measuring the expression of the inhibitor of NFκB α subunit (IκBα) which degradation is necessary for NFκB activation [51]. IκBα protein expression was similar in the three groups (Fig 2D). As inflammation and NFκB activation are related to IR development [52, 53], we assessed tissue insulin response by measuring basal and insulin-stimulated P-Akt/Akt protein levels in SAT biopsies. We did not observe any difference, neither for relative P-Akt/Akt level (Fig 3A) nor for insulin-stimulated fold induction of P-Akt/Akt (Fig 3B) between the three groups. Moreover, insulin-stimulated P-Akt/Akt fold induction in SAT did not correlate with the systemic insulin sensitivity-related indexes HOMA_IR_ (R = 0.108, P = 0.580; Fig 3C) and GIR (R = -0.281, P = 0.162; Fig 3D).

### Higher IκBα and blunted insulin response in skeletal muscle from OIR subjects

**Fig 4:**
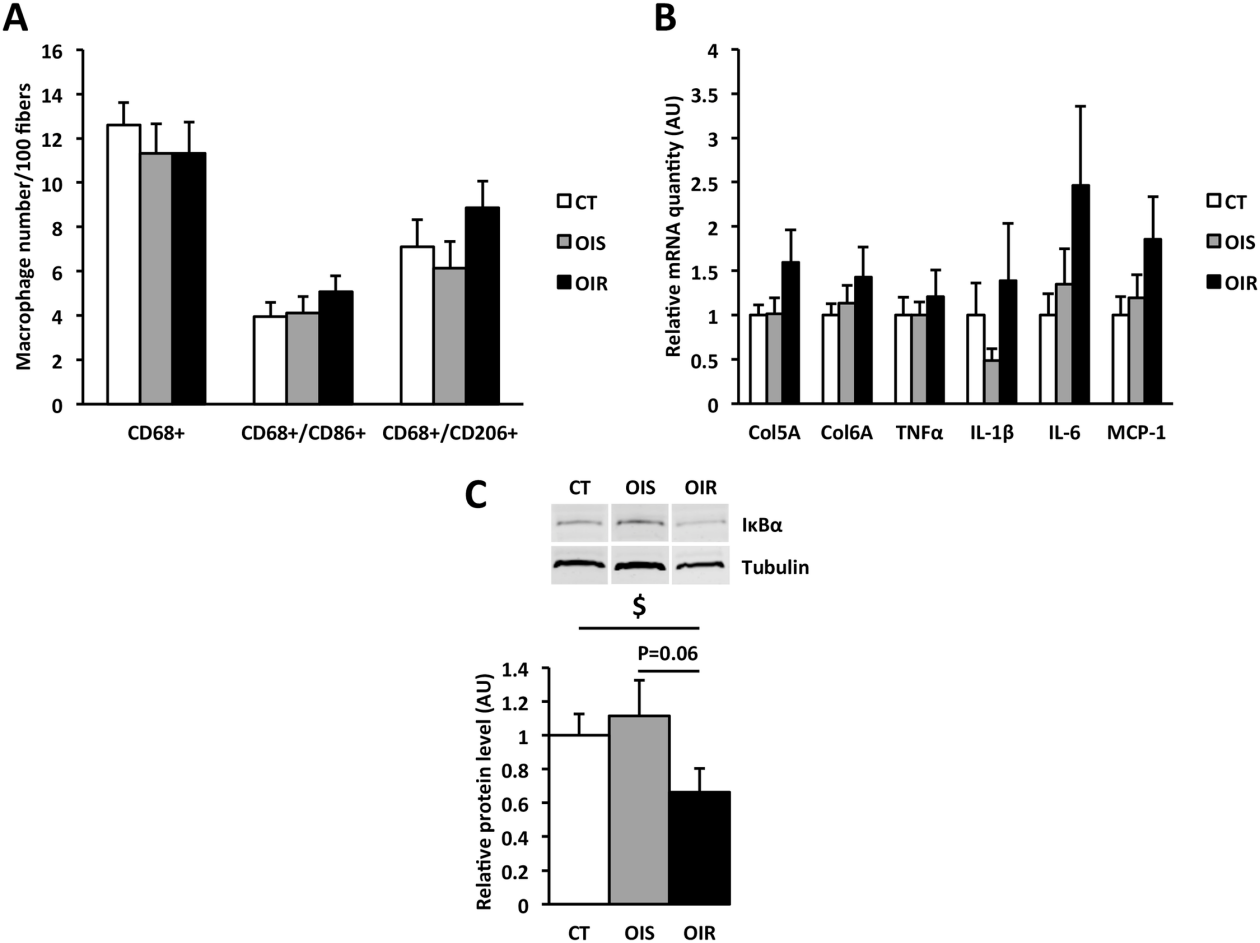
Inflammation and fibrosis in skeletal muscle. **Panel A:** Total macrophages (CD68^+^), and pro-inflammatory (CD68^+^/CD86^+^) and anti-inflammatory (CD68^+^/CD206^+^) macrophages were counted after staining in skeletal muscle. For CD68, and CD86 or CD206 markers, four and two non-consecutive entire sections per subject were respectively analyzed for 6 CT, 6 OIS and 5 OIR subjects. Data are expressed as mean ± SEM. **Panel B:** Relative quantification of Col5A, Col6A, TNFα, IL-1β, IL-6 and MCP-1, mRNA expression in skeletal muscle (n_CT_ = 10, n_OIS_ = 11, n_OIR_ = 9). Data are expressed as mean ± SEM and relatively to CT values, which were set at 1.0. **Panel C:** Western blot analysis of IκBα expression in skeletal muscle (n_CT_ = 10, n_OIS_ = 9, n_OIR_ = 9). The graph represents IκBα protein quantification after correction by α/β tubulin protein levels, used as an indicator of protein loading. Data are expressed as mean ± SEM and relatively to CT values, which were set at 1.0. $ P_OIR *vs*. CT_ = 0.04.

**Fig 5:**
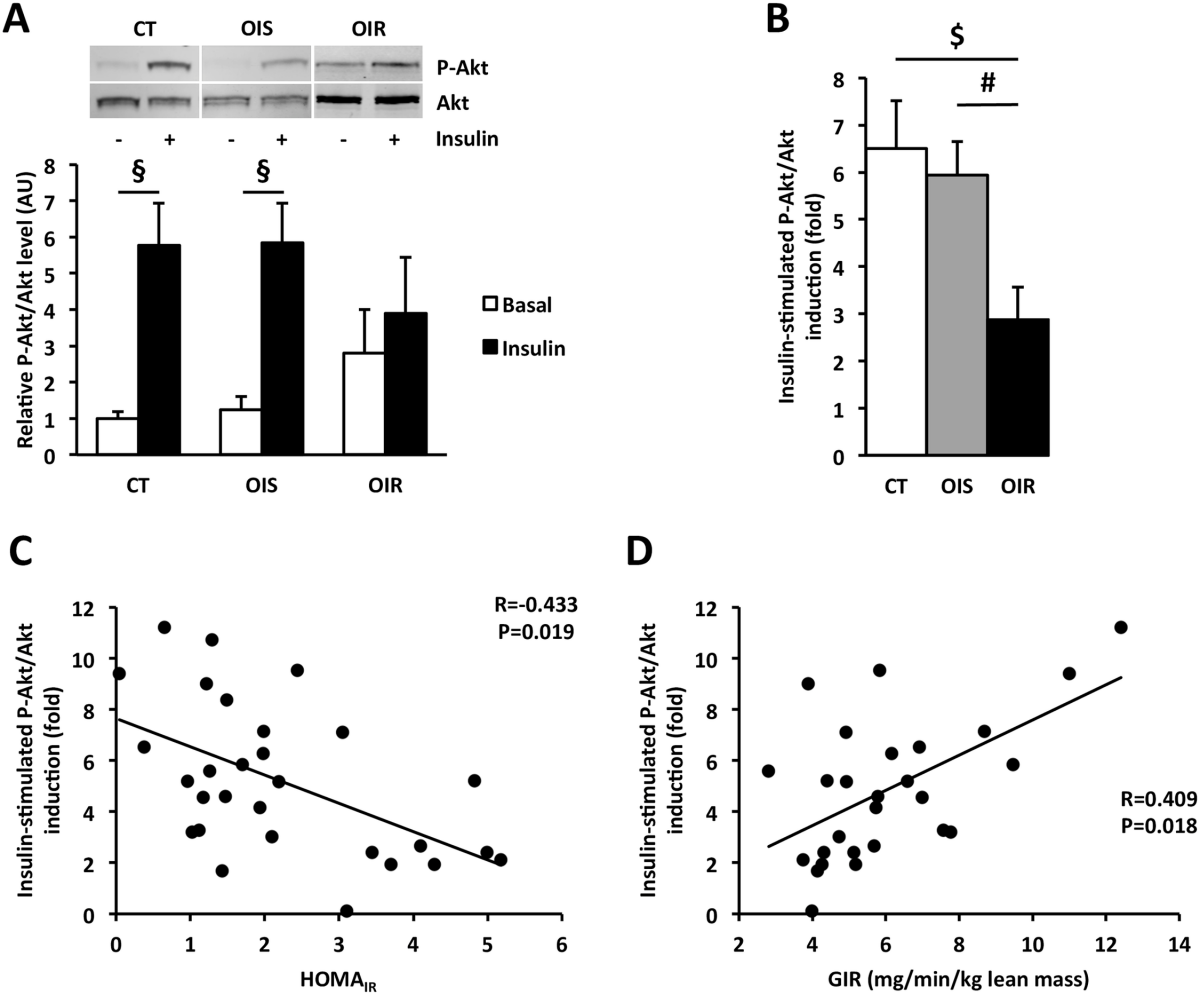
Insulin response in skeletal muscle. **Panel A:** Western blot analysis of P-Akt/Akt *ratio* in skeletal muscle with and without incubation with insulin (n_CT_ = 10, n_OIS_ = 11, n_OIR_ = 8). The graph represents P-Akt protein quantification after correction by total Akt protein levels, used as an indicator of protein loading. Data are expressed as mean ± SEM and relatively to CT values, which were set at 1.0. § P_CT±insulin_ = 0.002; P_OIS±insulin_ = 0.002. P_OIR±insulin_ = 0.129. **Panel B:** Fold induction of P-Akt/Akt in skeletal muscle after insulin stimulation. Data are mean ± SEM. $ P_OIR *vs*. CT_ = 0.008 and # P_OIR *vs*. OIS_ = 0.0005. **Panels C and D:** Correlations (Spearman analysis) between insulin-stimulated P-Akt/Akt fold induction in skeletal muscle and HOMA_IR_ (C) or GIR (D).

In this study, we also investigated whether inflammation developed locally within skeletal muscle. We did not observe any macrophage infiltration in skeletal muscle (S1 Fig) and counting of the different types of macrophages after staining revealed no difference in the number of total (CD68^+^), pro-inflammatory (CD68^+^/CD86^+^) or anti-inflammatory macrophages (CD68^+^/CD206^+^) between CT, OIS and OIR skeletal muscle (Fig 4A and S1 Fig). As for SAT, we did not observe any variation in Col5A and Col6A mRNA expression (Fig 4B), two subtypes of collagen which expression is modified in muscle IR [54]. The absence of local inflammatory response was supported by similar TNFα, IL-1β, IL-6, and MCP-1 mRNA expression in CT, OIS, and OIR skeletal muscle (Fig 4B). Regarding IκBα expression, we observed a lower level in OIR skeletal muscle compared to CT (P_OIR *vs*. CT_ = 0.04) and a tendency compared to OIS (P_OIR *vs*. OIS_ = 0.06) (Fig 4C). We also measured insulin-stimulated P-Akt/Akt protein levels to assess insulin response in skeletal muscle biopsies. In OIR skeletal muscle, contrary to CT and OIS, we observed no induction of P-Akt/Akt level following insulin stimulation (Fig 5A). Consequently, P-Akt/Akt fold induction was lower in OIR skeletal muscle compared to CT (P_OIR *vs*. CT_ = 0.002) and OIS (P_OIR vs. OIS_ = 0.002) (Fig 5B). Moreover, insulin-stimulated P-Akt/Akt fold induction in skeletal muscle was negatively correlated with HOMA_IR_ (R = -0.433, P = 0.019; Fig 5C) and positively correlated to GIR (R = 0.409, P = 0.018; Fig 5D), and we also found associations with IκBα protein expression (R = 0.525, P = 0.004) and systemic fetuin-A levels (R = -0.436, P = 0.018).

## Discussion

Systemic, SAT [2, 10, 11] and skeletal muscle [4, 8, 15] inflammation have been described as crucial determinant factors of IR and T2D development during obesity. To our knowledge, this study is the first comparing lifestyle behaviors, metabolic parameters and inflammation at both the systemic and tissue levels in normal-weight and obese subjects with different insulin sensitivity [30, 55].

The main findings of this study suggest that, in grade I obese post-menopausal women, muscle IR occurs prior to muscle and SAT inflammation and fibrosis, SAT IR and also prior to the development of a significantly measurable systemic inflammatory state.

Age, BMI, body adiposity index, and total fat mass were equivalent within the subgroups of obese subjects (Table 1), as were visceral adiposity index and waist circumference, two indexes indicative of abdominal/visceral fat distribution [56, 57]. However, although waist circumference was not significantly different between the obese groups, it was positively correlated to HOMA_IR_. This confirms that the increase in waist circumference is a strong risk factor of T2D and that the use of BMI-specific waist circumference cut-points [58] are more relevant to predict cardiometabolic risk than the threshold recommended by the current guidelines [59] (103 cm instead of 88 cm, respectively, for women with grade I obesity). In accordance with the literature, analysis of physical activity and diet composition showed that OIS and OIR subjects had similar overfeeding and sedentary behaviors (Fig 1, Table 1) [60, 61]. Likewise, several biological parameters such as lipids and adipokines were not different between OIS and OIR, whereas they differed between CT and OIS/OIR groups (see Tables 1 and 2). This suggests that these variations are linked to obesity and not to IR. In fact, apart from plasmatic insulin level, HOMA_IR_ and GIR, only diastolic blood pressure and ALT levels were altered in OIR subjects compared to OIS (Table 1), although remaining in normal range (diastolic blood pressure <90 mmHg and ALT<35 IU/L).

Regarding systemic inflammation, we did not observe any IR-associated systemic inflammation in this cohort. The pro-inflammatory cytokines TNFα, IL-1β, and IL-6, considered as highly involved in obesity-related IR [10, 21, 62] and the anti-inflammatory cytokines IL-4 and IL-10 [63] displayed similar levels in CT, OIS, and OIR subjects. Only hs-CRP level, although still in normal range (<7 mg/mL), was higher in OIS and OIR groups compared to CT. CRP has been well described as associated with obesity and several features of the metabolic syndrome [64]. Nevertheless, hs-CRP level was similar in OIS and OIR groups, in accordance with the observation that CRP levels did not differ among obese subjects with and without metabolic syndrome [65]. Moreover, in our study, hs-CRP levels were positively correlated with BMI and waist circumference. In humans, CRP is the major acute phase protein secreted by the liver due to IL-6 stimulation [66]. We thus hypothesized that liver-produced CRP might be induced by higher hepatic IL-6 concentration in OIS and OIR groups than in CT. However, we did not observe any significant systemic change for this cytokine. We can argue that, in contrast to the variability and instability of IL-6 level, the great stability and long half-life (19–20 hours) of CRP [67] might explain why we could measure higher systemic levels of hs-CRP in obese subjects without systemic IL-6 variation.

At the tissue level, our results do not support any role of SAT inflammation, fibrosis and/or IR in the development of systemic IR in post-menopausal women with grade I obesity. The absence of adipocyte hypertrophy in SAT from obese subjects leads us to consider a possible increase in adipocyte turnover. Indeed, when obesity develops, the excess lipid load can be compensated by enlargement of existing adipocytes and/or by increasing the normal process of adipocyte turnover in the adipose tissue [68]. Our results support the hypothesis that the recruitment of new adipose cells is required for expansion of body fat in obesity, particularly in SAT [69]. Indeed, McLaughlin and colleagues [70] recently showed that, for a 50% increase of body fat, the diameter of adipose cells increases by only 8% (for women) whereas the adipose cell number increases by 74%. In accordance with these data, there was no difference in adipocyte size between OIR, OIS and CT SAT. We did not observe any SAT fibrosis or M1/CD86^+^ macrophage infiltration in OIR SAT compared to CT and OIS. We could not even detect the particular subset of CD206^+^/CD11c^+^ inflammatory macrophages located in SAT crown-like structures and described by Wentworth and colleagues as associated with IR (data not shown) [47]. Of note, these macrophages were identified only in SAT from grade III obese women (BMI = 46±1 kg/m^2^) and not in women presenting a lower grade of obesity. On the contrary, we observed a slightly but significantly greater number of M2/CD206^+^ anti-inflammatory macrophages in OIR SAT compared to CT (Fig 2A), as also reported in another study [71]. This result is in agreement with the concomitant greater IL-6 mRNA expression in OIR SAT (Fig 2C). Indeed, IL-6 overexpression contributes in maintaining the anti-inflammatory macrophage population in adipose tissue that would limit the development of obesity-associated IR [72, 73]. In line with these results, IL-6 mRNA expression in SAT was positively correlated with M2/CD206^+^ macrophage number in SAT. In addition, we observed a similar insulin response in SAT biopsies from the three groups (Fig 3A and 3B). Our results are in favor of a positive role of IL-6, at least on inflammation and insulin sensitivity in SAT, in line with several other studies that showed a positive role of IL-6 on glucose metabolism [74–77]. This result was consistent with the absence of adipose tissue inflammation, fibrosis, and adipocyte hypertrophy that are generally closely related with the defect in insulin response [78, 79].

In skeletal muscle, M1/CD86^+^ and M2/CD206^+^ macrophage number (Fig 4A) and cytokine expression (Fig 4B) were similar in CT, OIS and OIR groups. We also observed a lower expression of IκBα in OIR skeletal muscle compared to CT and OIS (Fig 4C), indicative of TLR4 activation. However, we did not observe any variation in skeletal muscle TLR4 mRNA expression (data not shown) or in the systemic concentration of the TLR4 activators NEFA and LPS between CT, OIS and OIR. In accordance with the review from Karpe and colleagues [80], plasmatic levels of NEFA were in normal range for women after overnight fasting and unrelated to body fat mass and IR. LPS level was also similar in CT, OIS and OIR groups, as well as plasmatic levels of LBP and sCD14 (Table 2). Only the concentration of fetuin-A, an endogenous ligand of TLR4 required for its activation by NEFA [46], was greater in OIR group compared to OIS (Table 2), in accordance with previous studies [81, 82]. Interestingly, P-Akt/Akt protein *ratios* quantified from the muscle biopsies were positively correlated to IκBα protein expression and negatively correlated to systemic fetuin-A levels. These latter results suggest that impaired insulin response in OIR skeletal muscle might be due to higher levels of fetuin-A leading to increased activation of TLR4 signaling. This mechanism, which would require more investigations to be established, seems to occur only in skeletal muscle, as we did not observe such lower IκBα expression indicative of TLR4 activation in OIR SAT. In this study, we did not quantify intramyocellular lipids in muscle biopsies from CT, OIS and OIR subjects; therefore, we cannot exclude that the accumulation of toxic lipid intermediates within muscle fibers could participate in skeletal muscle IR. Indeed, several studies have previously reported that accumulation of diacylglycerols and ceramides play an important role in the development of muscle IR [83, 84].

As highlighted in this work by *in vivo* and *ex vivo* measurements of insulin sensitivity, skeletal muscle IR is an early event in obesity-related IR pathogenesis, which develops before any detectable macrophage infiltration in tissues, any SAT defect in insulin signaling or any systemic inflammation. In our cohort, muscle IR was highly associated with systemic IR, as shown by the correlations between the levels of insulin-stimulated P-Akt/Akt fold induction in skeletal muscle and the systemic insulin sensitivity-related indexes HOMA_IR_ and GIR. The absence of association between the levels of insulin-stimulated P-Akt/Akt fold induction in SAT and GIR was not surprising as during hyperinsulinemic-euglycemic clamp, the large majority of perfused glucose (85%) is used by skeletal muscle [13]. Therefore, GIR index mainly reflects skeletal muscle sensitivity to insulin. The strong correlation that we observed between muscle levels of insulin-stimulated P-Akt/Akt fold induction and HOMA_IR_ was in agreement with previous studies showing that HOMA_IR_ not only reflects hepatic insulin sensitivity but also peripheral and whole-body insulin sensitivity [85, 86]. However, we did not find any correlation between HOMA_IR_ and insulin-stimulated fold induction of P-Akt/Akt in SAT. The associations between insulin response, restricted to skeletal muscle, and the two indexes of systemic insulin sensitivity, HOMA_IR_ and GIR, stress the central role played by skeletal muscle in the regulation of whole-body insulin sensitivity.

Discrepancy between our observations and those previously described could be explained by differences in obesity severity of recruited subjects. In the literature, SAT inflammation was observed in morbidly obese (obesity grade III, BMI≥40.0 kg/m^2^) subjects. Macrophage infiltration is very progressive and usually occurs concomitantly with adipocyte hypertrophy, fibrosis, and obesity development [78, 79, 87]. Our cohort had grade I obesity and, although they already developed systemic IR, their SAT had not yet suffered from adipocyte hypertrophy and macrophage infiltration. Regarding macrophage infiltration in human muscle, this was observed in more severe obesity (grade II; BMI 35.0–39.9 kg/m^2^) [4] or in T2D patients [15, 88], which did not apply to our subjects who had even no personal or familial history of diabetes.

An essential strength of our study lies in the fact that the obese cohort we recruited was highly homogenous in terms of lifestyle and clinical and biological characteristics. This homogeneity enabled us to distinguish the parameters linked to obesity status and those linked to IR development. We could thus emphasize the central role played by skeletal muscle in IR development in low-grade obesity.

In conclusion, our results suggest that dysregulation of skeletal muscle insulin sensitivity is an early and central event of obesity-associated IR development and the principal feature to characterize insulin-resistant compared to insulin-sensitive post-menopausal women with grade I obesity. This finding may help to better understand the mechanisms involved in IR pathogenesis during obesity in human and personalize obesity clinical care to prevent the development of associated metabolic disorders such as T2D [89].

## Supporting Information

S1 Fig Macrophage staining in SAT and skeletal muscle. Representative immunohistochemical staining of pro-inflammatory macrophages (M1, CD86: red; total, CD68: green) and anti-inflammatory macrophages (M2, CD206: red; total, CD68: green) in SAT (Panel A) and skeletal muscle (Panel B). Nuclei were stained with DAPI in blue. On each image the arrow indicates one representative double-stained macrophage.
(TIF)

## Abbreviations

ALT: alanine aminotransferase
AST: aspartate aminotransferase
BAI: body adiposity index
BMI: body mass index
CT: controls (normal-weight insulin-sensitive subjects)
Col: collagen
DAPI: 4',6-diamidino-2-phenylindole
GGT: gamma-glutamyl transpeptidase
GIR: glucose infusion rate
HDLc: high-density lipoprotein cholesterol
HOMA_IR_: homeostasis model assessment of insulin resistance
hs-CRP: high-sensitivity C-reactive protein
IL: interleukin
IκBα: NFκB inhibitor α
IR: insulin resistance
LBP: LPS-binding protein
LDLc: low-density lipoprotein cholesterol
LPS: lipopolysaccharides
MD2: lymphocyte antigen 96
MCP-1: monocyte chemoattractant protein-1
NEFA: non-esterified fatty acids
NFκB: nuclear factor of kappa light polypeptide gene enhancer in B-cells
OIR: obese insulin-resistant
OIS: obese insulin-sensitive
SAT: subcutaneous adipose tissue
sCD14: soluble cluster of differentiation 14
T2D: type 2 diabetes
TLR: toll-like receptor
TNFα: tumor necrosis factor α
VAI: visceral adiposity index
