# Supplementary figures and images for: Skeletal Muscle Insulin Resistance and Absence of Inflammation Characterize Insulin-Resistant Grade I Obese Women
Source: PLoS One. 2016 Apr 25;11(4):e0154119. doi: 10.1371/journal.pone.0154119 (PMC4844150; doi:10.1371/journal.pone.0154119)

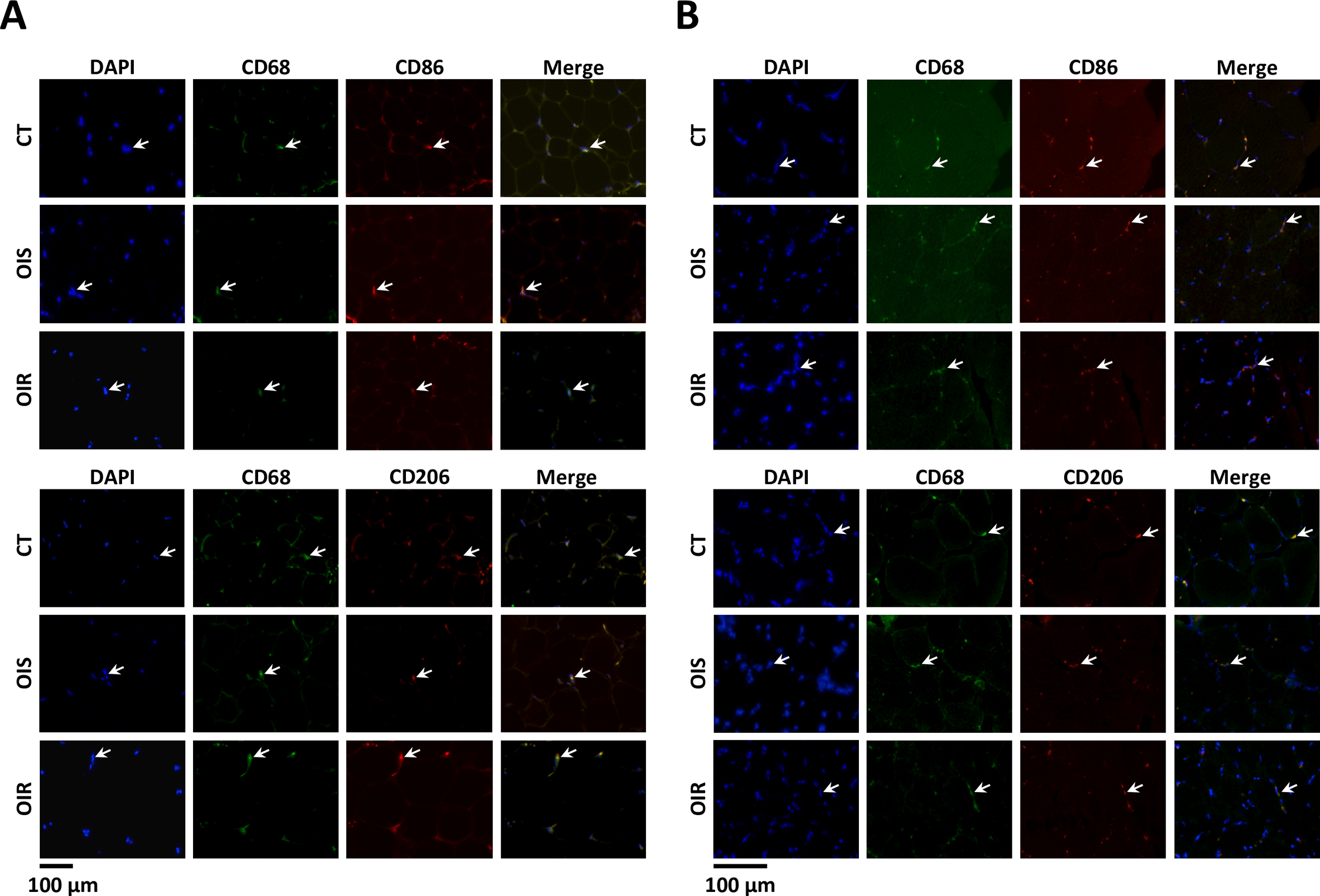

Supplement: S1 Fig — Representative immunohistochemical staining of pro-inflammatory macrophages (M1, CD86: red; total, CD68: green) and anti-inflammatory macrophages (M2, CD206: red; total, CD68: green) in SAT (Panel A) and skeletal muscle (Panel B). Nuclei were stained with DAPI in blue. On each image the arrow indicates one representative double-stained macrophage. (TIF) [file pone.0154119.s001.tif]
